# The value of ultrasound combined with CT in identifying early low-grade appendiceal mucinous neoplasm and appendicitis

**DOI:** 10.3389/fonc.2023.1191785

**Published:** 2023-10-02

**Authors:** Dong Bai, Nan Zhou, Ruixue Dou, Jiajun Wang, Pu Zhang, Haoyu Wang, Zhiqun Wang, Lei Liang

**Affiliations:** ^1^ Department of Radiology, Aerospace Center Hospital, Beijing, China; ^2^ Department of Ultrasound, Aerospace Center Hospital, Beijing, China; ^3^ Department of Myxoma, Aerospace Center Hospital, Beijing, China

**Keywords:** ultrasound, CT, low-grade appendiceal mucinous neoplasm, appendicitis, radiology

## Abstract

**Objective:**

The aim of this study is to investigate the value of ultrasound combined with computed tomography (CT) in identifying early low-grade appendiceal mucinous neoplasm and appendicitis.

**Methods:**

Patients with early low-grade appendiceal mucinous neoplasm and appendicitis from September 2017 to September 2021, including 40 patients with low-grade appendiceal mucinous neoplasm and 40 patients with appendicitis, were collected in this study. Clinical data as well as ultrasound and CT findings of all patients were retrospectively analyzed. Univariate and multivariate logistic regression analyses were applied to establish the ultrasound model, the CT model, and the combined model.

**Results:**

The nomogram showed that specific characteristics of CT were dilated appendiceal diameter and clear surrounding fat space in the low-grade appendiceal mucinous neoplasm and that specific characteristics of ultrasound were thin or clear layer appendix wall and flocculent echo in the appendix cavity. These four features were used to construct a nomogram for predicting early low-grade appendiceal mucinous neoplasm, and the area under the curve value was 0.839.

**Conclusion:**

Ultrasound combined with CT for diagnosis of early low-grade appendiceal mucinous neoplasm has a significant value; when found significantly dilated appendix in the lower right abdomen, with thin wall, wall calcification, clear surrounding fat space, and progressive enhancement, especially non-specific symptoms similar to appendicitis, the physician should timely consider the possibility of low-grade appendiceal mucinous neoplasm.

## 1 Introduction

Appendiceal mucinous neoplasm is a rare disease with an incidence of less than 1% (1) in all appendectomy specimens. According to the latest 2019 version of the WHO classification, appendiceal mucinous neoplasm is divided into low-grade appendiceal mucinous neoplasm (LAMN) and high-grade appendiceal mucinous neoplasm (HAMN) (2).

The onset of LAMN is insidious and lacks specific clinical manifestations. It often presents with lower right abdominal pain, lower right abdominal mass, and abdominal distension as the first symptoms. It is often misdiagnosed as appendicitis preoperative and removed laparoscopically; finally, it is diagnosed through intraoperative or postoperative pathology (3). Improper handling easily causes rupture planting; lately, patients found as pseudomyxoma peritonei (PMP) because of abdominal distension symptom. Thus, they missed the treatment opportunity, and it affected the survival rate of 5 years. Therefore, early diagnosis and surgery of LAMN, the preoperative and intraoperative prevention of tumor rupture, and the prevention of tumor implant and metastasis are the key (4) to improving patient survival. With the development of imaging technology, at present, the most common examination methods applied to LAMN are mainly ultrasound (US) and abdominal computed tomography (CT) plain scan plus enhancement, and some scholars also use magnetic resonance and PET-CT to diagnose this disease. In the future, we will consider comparing the diagnostic value of multiple imaging methods in this disease, so as to find the optimal combination of imaging methods and realize multi-center research as much as possible.

Most of the literature focused on the case reports of LAMN; this study collected 40 patients with LAMN, combined with clinical data from US and CT, to analyze the clinical and imaging characteristics of early LAMN and appendicitis by nomogram. It aims to summarize the characteristic indicators of early LAMN and to provide a reliable basis for the diagnosis of early LAMN. By describing these diseases, we not only can enhance clinical understanding of the disease but also can improve patient outcomes.

## 2 Materials and methods

### 2.1 Study population

This study was a retrospective analysis from a single study center. The Hospital Ethics Review Board approved it, and all study subjects were informed about and consented to enter the study. Patients with early LAMN and appendicitis confined by surgical pathology from September 2017 to September 2021 were selected.

### 2.2 Clinical data

Clinical data of all patients were collected, including age, sex, clinical symptoms, white blood cell (WBC) number, carcinoembryonic antigen (CEA), and C-reactive protein (CRP).

### 2.3 Image and data

All the patients had CT plain scan and US examination before surgery, and all the patients with LAMN underwent CT enhancement examination. Intestinal preparation method for CT examination: fasting (fasting water for 8 h). On the day of examination, gastrointestinal imaging agent (iodide) was orally administered three times within 2 h before taking the machine, 500 mL each time. Scanning was performed by a SOMATOM Siemens Force dual-source CT device. Conventional supine position, the scanning area from the top of the diaphragm to the iliac spine level, enhanced scanning using a high-pressure syringe through antecubital intravenous injection of nonionic contrast agent (iddfool or iodopadol) 75~100 mL, injection speed 2.5~3.0 mL/s, tube voltage of 120 kV, tube current of 200~250 mA, matrix of 512 × 512, pitch of 1.0, and layer thickness and spacing of 5 mm. The images were reconstructed in 1.25-mm axial thin-layer, coronal, and sagittal planes. US examination was using an Aixplorer^®^ US scanner (SuperSonic Imagine, Aix en Provence, France), with low-frequency and high-frequency probes; frequency values were 3–5 MHz and 9–12 MHz, respectively. The patient was taken in the conventional supine position, scanned with the high-frequency and low-frequency probes, and the images of the appendix size (long diameter and outer diameter), the appendix wall thickness, the appendix wall hierarchy and continuity, the echo in the appendix cavity, and the surrounding appendix were collected.

Two radiologists with more than 5 years of radiology experience read CT images, double- blind for imaging diagnosis and characteristics of these images, including appendix length, luminal diameter, wall thickness, wall calcification, intraluminal density, surrounding effusion, enlarged lymph nodes, wall density, adjacent fat space, and fecal stones. The US images were also read double-blind by two US physicians with more than 5 years of US diagnosis experience; appendix length, luminal diameter, wall thickness, wall hierarchy, intraluminal echo, enlarged lymph nodes, surrounding effusion, and omentum parcel were evaluated. The interval between US and CT and surgery is 2–5 days.

### 2.4 Statistical analysis

Univariate and multivariate logistic regression analyses were applied to establish the US model, the CT model, and the combined model. Stepwise regression forward method was used in logistic regression analysis. The area under the curve (AUC) was used to quantify the performance of each model. The nomogram of the combined model was constructed to improve decision-making. Statistical analysis was performed using the R software (version 4.0.2) and SPSS (version 23.0) software.

## 3 Results

### 3.1 Patient characteristics

The characteristics of all patients with LAMN and appendicitis are shown in **Table 1**. The mean age of the patients with LAMN was 59 years old, and the male-to-female ratio was 1.2:1. The mean age of patients with appendicitis was 46 years old, and the male-to-female ratio was 1.1:1. Clinical symptoms of patients with LAMN are as follows: 21 patients experience lower right abdominal pain, 13 patients were initially suspected of acute appendicitis, 13 patients were asymptomatic due to physical examination, six patients abdominal distension. Clinical symptoms of patients with appendicitis are as follows: All patients experience lower right abdominal pain. Laboratory examination showed that one patient with LAMN had mildly elevated leukocytes and that six patients tumor markers of CEA were slightly increased. However, 35 patients with appendicitis had WBC increased, and CEA was not increased.

**Table 1:** Clinical characteristics analysis of the patients with LAMN and appendicitis.

Characteristics	LAMN	Appendicitis	Univariate logistic analysis	Multivariate logistic analysis
			p-value	p-value
No. of patients, n	40	40		
Gender, n			0.823	
Male	22	21		
Female	18	19		
Age, years	58.9 ± 9.8	46.5 ± 19.6	0.002*	0.114
CEA (ng/mL)	3.8 ± 3.6	1.44 ± 0.6	0.000*	0.428
WBC (10^9^/L)	6.2 ± 1.5	10.7 ± 3.4	0.000*	0.180
CRP (mg/L)	7.6 ± 6.6	85.0 ± 81.5	0.000*	0.387

"*" means P<0.05.

### 3.2 Ultrasound characteristics

**Figure 1:**
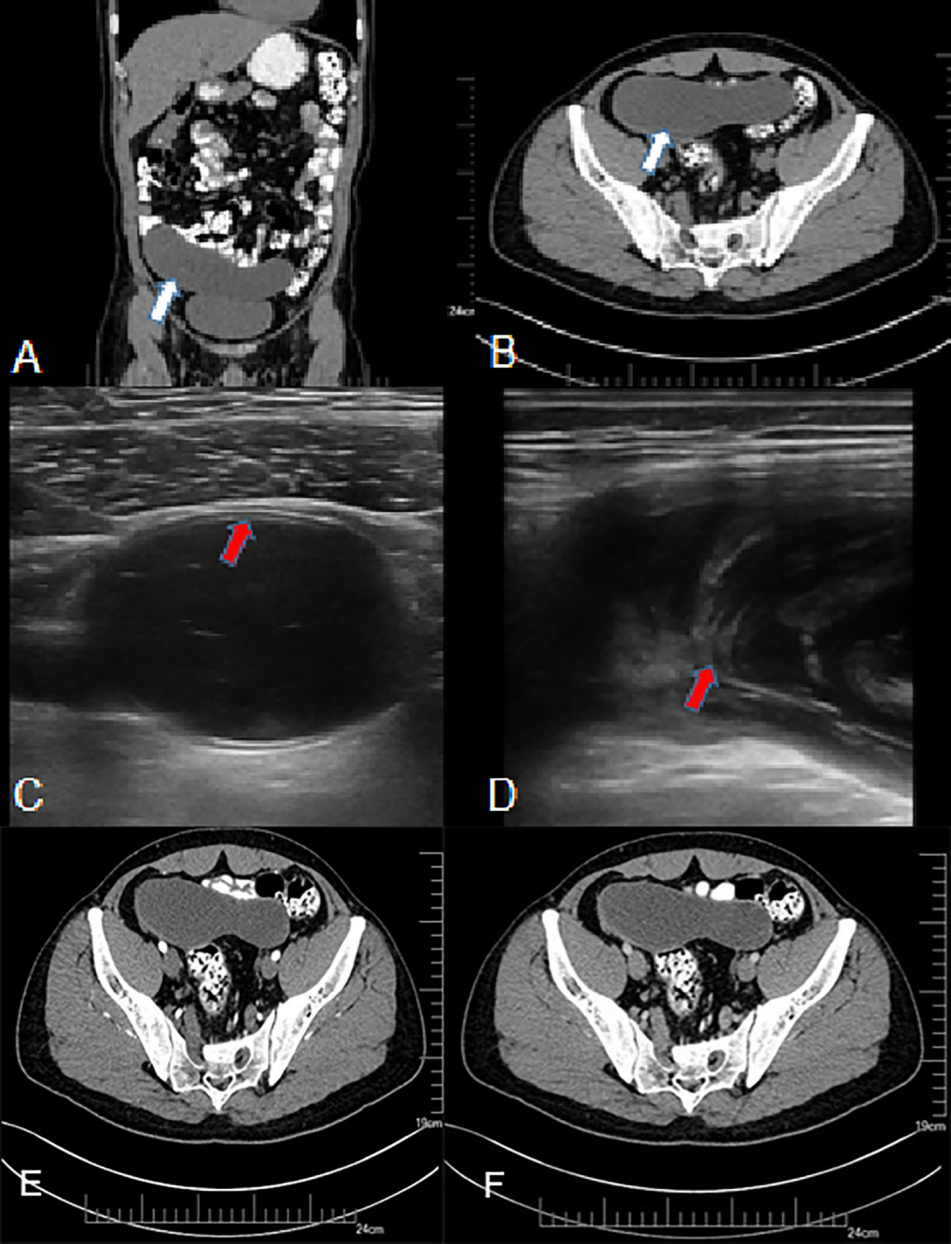
A 50-year-old man with LAMN. **(A, B)** Plain CT scan, showing significant widening of the appendix, a thin-walled low-density focus (white arrow), with uniform density and clear surrounding fat space. **(C, D)** US examination, **(C)** showing clear layer of the appendix wall (red arrow) and **(D)** showing flocculate echo in the appendix cavity (red arrow). **(E, F)** CT enhancement; after enhancement, there was no clear enhancement in the center of the lesion, and mild delayed enhancement was observed at the edge.

Typical US features of LAMN included appendix swelling, thin walls, clear wall layers, and flocculus echo in the appendix cavity (**Figure 1**). However, US of appendicitis presented with thick appendix wall, unclear wall layers, hypoechoic in the appendix cavity, wrapping the surrounding omentum, and enlarged lymph nodes and effusion around appendix (**Table 2**).

**Table 2:** Univariate and multivariate analysis of ultrasound characteristics.

Characteristics	LAMN	Appendicitis	Univariate logistic analysis	Multivariate logistic analysis
			p-value	p-value
Length, cm	6.7 ± 2.9	5.4 ± 1.4	0.018*	0.207
Luminal diameter, cm	2.5 ± 1.1	1.0 ± 0.3	0.000*	0.471
Wall thickness, mm	2.3 ± 0.6	4.1 ± 0.8	0.000*	0.007*
Wall layers, n			0.000*	0.349
Clear	35	12		
Not clear	5	28		
Intraluminal echo, n			0.000*	0.016*
Echoless	2	40		
Flocculent echo	38	0		
Omentum parcel, n			0.993	
No	35	10		
Yes	5	30		
Surrounding effusion, n			0.990	
No	40	22		
Yes	0	18		
Enlarged lymph nodes, n			0.992	
No	40	35		
Yes	0	5		

"*" means P<0.05.

### 3.3 CT characteristics

Typical CT features of LAMN showed thin-walled low-density focus with smooth cystic walls, uniform intraluminal density, mostly with wall calcification, clear surrounding fat space, mild progressive enhanced cystic wall, and intact cystic walls (**Figure 1**). The image of appendicitis showed thickening and coarse appendix wall, blurred surrounding fat space, uneven density in the cavity, with gas and fecal stones, surrounding effusion, and enlarged lymph nodes (**Table 3**).

**Table 3:** Univariate and multivariate analysis of CT characteristics.

Characteristics	LAMN	Appendicitis	Univariate logistic analysis	Multivariate logistic analysis
			p-value	p-value
Length, cm	6.4 ± 2.9	5.6 ± 1.6	0.164	
Luminal diameter, cm	3.2 ± 2.1	1.1 ± 0.4	0.000*	0.006*
Wall thickness, mm	2.0 ± 1.3	4.3 ± 1.1	0.000*	0.494
Wall calcification, n			0.993	
Yes	24	0		
No	16	40		
Intraluminal density, n			0.823	
Uniform	23	11		
Non-uniform	17	29		
Surrounding effusion, n			0.992	
No	40	14		
Yes	0	26		
Enlarged lymph nodes, n			0.990	
No	40	32		
Yes	0	8		
Wall density, n			0.008*	0.740
Uniform	27	0		
Non-uniform	13	40		
Fat space, n			0.000*	0.000*
Blurred	1	40		
Clear	39	0		
Fecalith, n			0.989	
Yes	0	29		
No	40	11		

"*" means P<0.05.

### 3.4 Pathological characteristics

**Figure 2:**
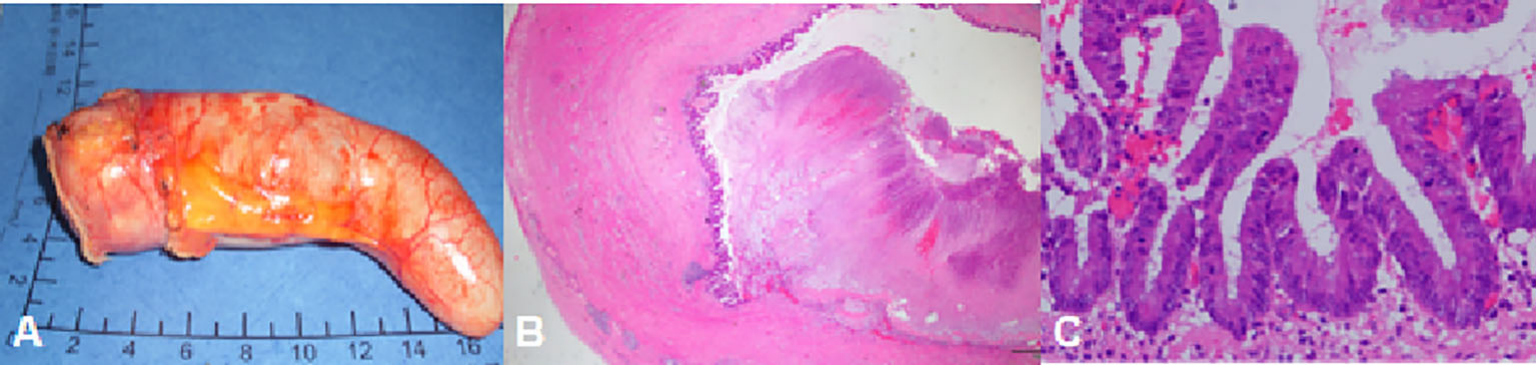
A 50-year-old man with LAMN. The pathology showed complete fibrosis in part of the appendix wall and no tumor in the serosal.

The pathology of LAMN was partial muscle wall fibrosis, with acellular mucus in the submucosal layer and between the fibrous walls. There were no lesions in the serosal, and the appendix was not ruptured (**Figure 2**).

In this study, 10 cases were acute simple appendicitis: Inflammation was limited to the mucosa and submucosa; 23 cases of acute suppurative appendicitis: inflammation involving all layers of the appendix, local purulent, and necrosis; seven cases were acute gangrene appendicitis: extensive bleeding necrosis in all layers of the appendix.

### 3.5 Nomorogram

**Figure 3:**
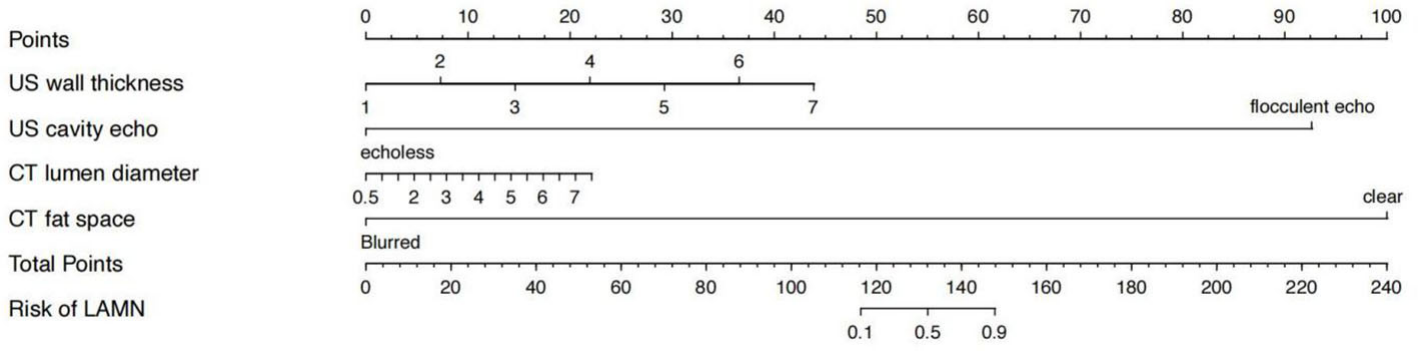
Nomogram for predicting early low-grade appendiceal mucinous neoplasm.

**Figure 4:**
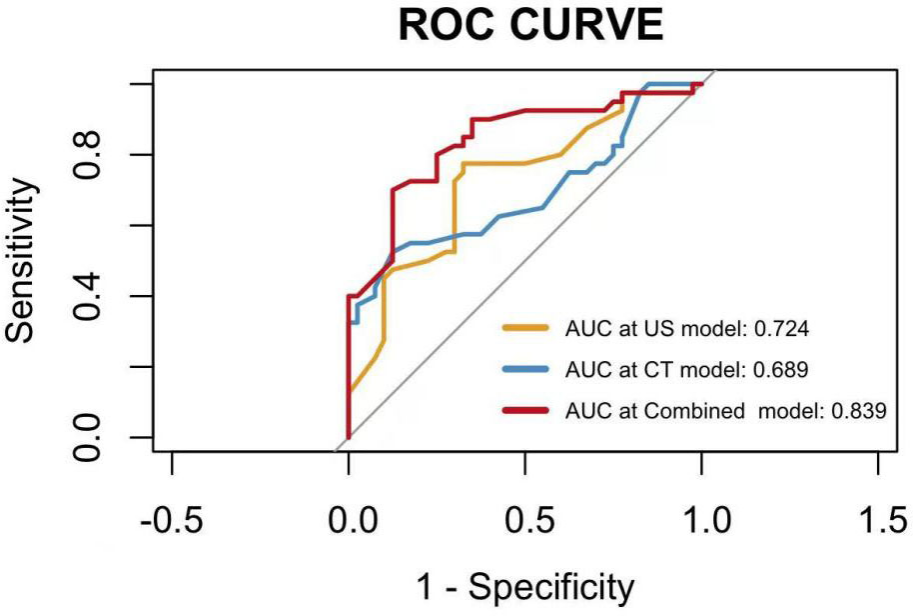
The ROC curves of the ultrasound model, the CT model, and the combined model.

The nomogram showed that specific signs of CT were dilated appendiceal diameter and clear surrounding fat space in the LAMN, and specific signs of US were thin- or clear-layer appendix wall and flocculent echo in the appendix cavity. These four features were used to construct a nomogram for predicting early LAMN (**Figure 3**); the Receiver Operating Characteristic (ROC) curve shows that the AUC values of the US model, the CT model, and the combined model were 0.724, 0.689, and 0.839, respectively (**Figure 4**).

## 4 Discussion

The appendiceal mucus cyst, first described by Rokitansky, showed mucus accumulation in the appendiceal cavity and progressive expansion (5). Higa et al. suggested that all mucinous cysts were mainly due to the following causes: retention cysts (1%), mucosal dysplasia (25%), mucinous cystadenoma (63%), and mucinous cyst carcinoma (11%) (6). However, this classification does not provide a realistic view of the lesions. To address this problem, in 2016, the International Group of Peritoneal Surface Oncology Group International divided appendiceal mucinous neoplasm into adenomas or serrated lesions, LAMN, HAMN, and mucinous adenocarcinomas (7). Low-grade mucinous neoplasm is mucinous neoplasm with low-grade atypical epithelial hyperplasia. The epithelium is advanced into muscularis propria mucosa, restricted by muscularis propria, is non-invasive, and lacks connective tissue response (8). In 2019, the WHO classified most of the appendicular noninvasive mucinous neoplastic lesions as low-grade mucinous neoplasm (9).

Low-grade mucinous neoplasm of the appendix had been reported in the literature for a female-to-male ratio of 4:1 and tends to affect patients over 50 years of age (10). However, the male-to-female ratio of this study was 1.2:1, and the average age was 59, which was inconsistent with literature reports. The analysis may be related to the small sample size of this study. The most common clinical presentation of LAMN is pain in the right iliac fossa, similar to acute appendicitis. However, about 51% of patients are asymptomatic, and their condition is discovered by chance during imaging or surgery (11). Other complications of the disease include intestinal obstruction, intussusception, gastrointestinal bleeding, and extrinsic ureteral compression, leading to hydrops in the urinary tract and increasing abdominal circumference (12). Consistent with literature reports, the main clinical symptom of LAMN patients in this study was the lower right abdominal pain. The most serious complication of LAMN is peritoneal pseudomyxoma secondary to spontaneous or iatrogenic rupture of the appendix and consequent overflow of tumor cells and mucins into the peritoneal cavity (13). It has been confirmed that LAMN is the most common cause of low-grade PMP because the risk of lymph node or hematogenous transmission is very low (14). However, it is easily misdiagnosed as appendicitis and other diseases; patients are often diagnosed with the disease during intraoperative or postoperative pathology, resulting in iatrogenic destruction of the cyst wall and the spread of mucus into the abdominal cavity. Therefore, it is necessary to identify low-grade mucinous neoplasm confined to the appendix, and early surgical complete resection of the disease can improve the 5- or even 10-year survival rate of patients.

This study also reviewed clinical laboratory indicators, including WBC, CRP, and CEA. Among them, WBC and CRP are the commonly used laboratory indicators for the diagnosis of acute appendicitis. They are not only simple to operate but also have low testing costs (15). When an inflammatory reaction occurs, a large number of leukocytes are immediately released into the blood under the action of chemokines. Experiments have shown that peripheral blood WBC count and CRP are the best laboratory methods for diagnosing acute appendicitis. However, because there are still some patients whose peripheral blood WBC count and CRP are not elevated, and they are affected by many other factors, even in patients with severe infection, there are still a small number of patients whose WBC count and CRP are not elevated and their sensitivity and specificity are low (16).

The serum tumor marker CEA is widely used in the diagnosis, prognosis evaluation, curative effect, and recurrence monitoring of gastric and colorectal cancer. However, the diagnostic value of these tumor markers for primary appendix tumors is unclear (17). Pablo et al. reported that CEA was mostly within the normal range in patients with early LAMN, and the increased levels were mainly seen in patients with advanced stage and recurrence (18). The results of this study were consistent with its findings.

CT has proven to be the fastest and most accurate method, with an accuracy rate of 89.7% (19). On CT imaging, the normal appendix appears as a thin-walled tubular or annular structure surrounded by lower right abdominal mesenteric fat, generally <6 mm in diameter. This study showed that CT of LAMN mainly showed oval and tubular low-density lesions in the lower right abdomen with smooth edges. The diameter of the lumen ranged from 1.4 cm to 11 cm, with an average diameter of about 3.2 cm. Calcification was observed in the cyst wall in 45% of the patients, and mild enhancement was observed in the cyst wall after enhancement, with clear surrounding fat space and no obvious enlarged lymph nodes.

In this study, the nomogram showed CT-specific signs of LAMN, including widening of the appendiceal lumen and clear fat space around the appendix. LAMN is caused by abnormal accumulation of mucus caused by adenomatous hyperplasia of the appendiceal epithelium, which leads to obvious expansion of the lumen. Pathologists point out that LAMN presents pushing growth (20), which is morphologically manifested as expansion of the lumen of the appendix. Appendiceal myxoma and mucinous adenocarcinoma of the appendix have been reported in the past to have a diameter of up to 6 cm, and some cases reported a diameter of up to 10 cm (21–23). In this study, the average diameter of the appendix of the 40 patients with LAMN was about 3.2 cm, and the maximum diameter was about 11 cm, which was consistent with the reports in the literature. The diameter of the appendicitis in the control group ranged from 0.6 cm to 2.5 cm, the average diameter was about 1.1 cm, and the difference was statistically significant.

In this study, all LAMN cyst walls were smoothed, except for one case with blurred peripheral fat space complicated with acute appendicitis, and the rest of the lesions had clear peripheral fat space. The cystic wall was encapsulated by pathological hyalinization and fibrosis. The average thickness of the cystic wall was about 2.0 mm, whereas the average thickness of the cystic wall in appendicitis was about 4.3 mm. The cyst wall was thickened and coarse. Because of inflammatory stimulation, the fat space around the appendix was blurred, and water-like effusion was seen around 60% of the patients, which was consistent with literature reports (24). The density in LAMN appendiceal cavity was uniform, the CT value range was about 15–29 hounsfield unit (HU), and there was no bezoite in the appendiceal cavity. Appendicitis is caused by various causes of inflammatory lesions, the most common cause is the appendix cavity fecalith obstruction, and the density of the appendix cavity is mixed; gas accumulation can be seen in some, so they can be distinguished from each other to a certain extent. In this study, patients with LAMN underwent enhanced examination, and patients with appendicitis only underwent plain CT examination. The LAMN capsule wall showed mild enhancement in the arterial phase after enhancement, and progressive enhancement was seen in the portal vein and delayed phase due to fibrosis of the capsule wall. There was no clear hyperenhancing nodule in the cystic cavity, and it has been reported in the literature that the hyperenhancing nodule in the cystic cavity is highly suggestive of mucinous adenocarcinoma (25). Pathologically, LAMN shows fibrosis, hyaline degeneration, and calcification in the cyst wall, so curvilinear calcifications in the cyst wall are highly suggestive of LAMN, and calcification has nothing to do with the degree of malignancy (24, 26). This study is consistent with the literature reports, but no clear cyst wall calcification was found in appendicitis lesions, and the analysis of LAMN cyst wall calcification may be caused by long-term stimulation of cystic mucus.

US, especially the application of high-frequency US, has unique advantages in the diagnosis of LAMN and appendicitis. The nomogram in this study showed that the US-specific signs of LAMN included thin appendix wall and flocculated echoes in the appendix cavity. These signs are also superior to US over CT.

Some pathological studies have shown that the appendiceal wall of some cystic dilated appendiceal lesions often appears as thin fibrous membrane, and the normal structure of the appendiceal wall disappears (27). High-frequency US has unparalleled advantages in observing the layers of the intestinal wall and can clearly display the light-dark-bright three-layer structure of the appendix wall. In this study, most of the early LAMN appendix walls were thinner than those of patients with appendicitis, and the structure was clear. In some cases, thin fibrous membrane was formed in the local appendiceal wall, showing a strong linear echo. The analysis suggests that LAMN tumor epithelial cells are low level and less invasive, so they have not damaged the appendiceal wall in the early stage. However, if it is allowed to develop, then once the ruptured appendiceal tumor cells spread outside the appendiceal cavity, it indicates a poor prognosis (28). It can be seen that high-frequency US has important value in the identification and diagnosis of appendix lesions in observing the layers and continuity of the appendix wall.

This study found that all patients with early LAMN had flocculent echoes in the appendix cavity; because LAMN tumor cells are rich in mucin and can secrete a large amount of mucus, the mucin-to-cell ratio can be as high as 1:1000, which is relatively viscous (29). US examination, especially high-frequency US, has unique advantages in the identification of liquid components. Such mucus appears flocculent on sonography, which is an important feature of LAMN to identify appendicitis.

The results of this study showed that the diagnostic performance of CT combined with US was significantly better than that of CT alone or US alone in the diagnosis of LAMN and appendicitis. The main advantage of CT is fast thin-section scan, which is less affected by intestinal gas and operation factors, especially for the widening of the appendix cavity, the calcification of the capsule wall, and the change of the surrounding fat space. US examination is convenient and non-radioactive and has unique advantages in the level of appendix wall and the echo characteristics of the appendix cavity. Therefore, the two examinations can complement each other and provide more evidence for the early diagnosis of LAMN. In addition, there are literature reports that PET-CT is also valuable in the diagnosis of this type of disease. Li et al. found that, on PET-CT images, LAMN group showed shorter diameter and lower cystic wall metabolic level [maximum standardized uptake value (SUV max) 2.73 ± 1.31] than those of mucinous appendiceal adenocarcinoma group. 18F-FDG PET-CT examination has a good diagnostic value (30).

The main innovations of this study are as follows: First, the combination of US and CT imaging examination, combined with the advantages of both, can make the diagnosis of early LAMN more comprehensive and accurate; secondly, the nomogram is convenient for clinicians to make quick and simple clinical diagnosis and decision-making. The limitations of this study mainly include the following: first, LAMN is rare, and the sample size of this study is small; second, there is a bias in patient selection, the retrospective design of the analysis was based on pathology rather than imaging findings of appendiceal abnormalities, and the study population was limited to a single center study.

LAMN is very rare and can be confused with acute appendicitis. The most important diagnostic tools are US and CT. Preoperative diagnosis of LAMN is very important for surgical methods and prognosis, so as to avoid the development of peritoneal pseudomyxoma. If imaging shows tubular hypoechoic or low-density dilated appendix in the lower right abdomen, thin wall, clear surrounding fat space, calcification in the cyst wall, and progressive enhancement after enhancement, especially non-specific symptoms similar to appendicitis in middle-aged and elderly women, then the possibility of LAMN should be considered in time. This disease is very rare in clinic, and many basic hospitals lack the understanding of this kind of disease. Through this study, in addition to summarizing some clinical and imaging characteristics, this paper also hopes to popularize and publicize this disease and to improve the awareness of hospitals at all levels. Although preoperative diagnosis is still challenging, we point out that the combination of clinical manifestations and US and CT imaging features can significantly improve the diagnostic accuracy of LAMN, thus providing an important basis for surgeons to make preoperative surgical planning.

## Data availability statement

The original contributions presented in the study are included in the article/supplementary material. Further inquiries can be directed to the corresponding authors.

## Ethics statement

The studies involving humans were approved by The Hospital Ethics Review Board. The studies were conducted in accordance with the local legislation and institutional requirements. Written informed consent for participation was not required from the participants or the participants’ legal guardians/next of kin in accordance with the national legislation and institutional requirements.

## Author contributions

LL and ZW conceived and designed the experiments; DB and NZ equally contributed to this work, and they jointly completed the study design, data collection, statistical analysis, and article writing; RD, PZ, and HW mainly completed the data collection work; JW completed the data analysis and chart production. All authors contributed to the article and approved the submitted version.
